# Comprehensive Analysis of Human Colorectal Cancers Harboring Polymerase Epsilon Mutations

**DOI:** 10.3390/ijms26157208

**Published:** 2025-07-25

**Authors:** Louis M. Gibson, Phanithan Konda, Hunter J. Bliss, Devi D. Nelakurti, Golrokh Mirzaei, Renee A. Bouley, Jing J. Wang, Ruben C. Petreaca

**Affiliations:** 1 Neuroscience Undergraduate Program, The Ohio State University, Columbus, OH 43210, USA; lgibson1224@outlook.com; 2 The James Comprehensive Cancer Center, The Ohio State University, Columbus, OH 43210, USA; devidheekshita.nelakurti@osumc.edu; 3 College Credit Plus Program, The Ohio State University, Columbus, OH 43210, USA; 4 Biology Undergraduate Program, The Ohio State University, Marion, OH 43302, USA; bliss.92@buckeyemail.osu.edu; 5 Biomedical Science Undergraduate Program, The Ohio State University College of Medicine, Columbus, OH 43210, USA; 6 Department of Computer Science and Engineering, The Ohio State University, Marion, OH 43302, USA; mirzaei.4@osu.edu; 7 Department of Chemistry and Biochemistry, The Ohio State University, Marion, OH 43302, USA; bouley.8@osu.edu; 8 Department of Cancer Biology and Genetics, The James Comprehensive Cancer Center, The Ohio State University Wexner Medical Center, Columbus, OH 43210, USA; 9 Department of Molecular Genetics, The Ohio State University, Marion, OH 43302, USA

**Keywords:** colorectal cancer, polymerase epsilon, driver mutation

## Abstract

DNA polymerase epsilon (POLe) is the leading strand replicative polymerase. POLe mutations located primarily in the proofreading domain cause replication errors and increase mutation burden in cancer cells. Consequently, POLe has been classified as a cancer driver gene. Certain POLe frameshift mutations that affect the proofreading domain are purified in cancer cells, but point mutations in other domains have also been reported. Here we use an artificial intelligence algorithm to determine what other mutations co-occur with POLe mutations in colorectal cancers. We partitioned POLe mutations into driver, passenger, and WT (no mutation), then assessed mutations in other genes in these three groups. We found that a driver POLe mutation is not likely to associate with driver mutations in other genes. Thus, driver mutations in colorectal cancers appear to purify in a manner that is independent of POLe. Mutations that affect POLe function do not necessarily increase the frequency of driver mutations in other genes. Structural analysis shows that many POLe driver mutations affect coordination of the Mg^2+^ ion in the active site. Our data show that the accumulation of colorectal cancer mutations is driven by complex factors.

## 1. Introduction

Colorectal cancer (CRC) is the third most common cancer and second leading cause of cancer mortality in the US, with the majority of deaths from metastatic disease. While the 5-year survival rate for localized CRC is approximately 90%, it drops to 14% when patients present with distant metastases (https://www.cancer.org/cancer/types/colon-rectal-cancer/detection-diagnosis-staging/survival-rates.html (accessed on 16 July 2025)). Treatment of metastatic disease with chemotherapy or targeted drugs has not had a large clinical impact, in part because CRC is a disease associated with a high degree of heterogeneity, leading to complicated phenotypes (https://www.cancer.org (accessed on 16 July 2025)).

Genome instability is a hallmark of cancer and a major feature of CRC [1,2]. Integrated molecular analysis that includes a variety of genomic and transcriptomic data has classified CRC into non-hypermutated, hypermutated, and ultramutated groups [3]. The non-hypermutated group that comprises the majority (~80%) of CRC presents with ˂10 mutations/million basepairs (Mb) and stable but polymorphic simple repeat sequences (microsatellite stability or MSS). MSS tumors often display a high frequency of DNA copy number alterations resulting from sequence gains/deletions, translocations and other chromosomal rearrangements [2]. The hypermutated group accounts for 13–15% of CRC, has 10–100 mutations/Mb, and exhibits microsatellite instability (MSI), principally due to defective mismatch repair (MMR). The ultramutated group displays ˃100 mutations/Mb and accounts for ~3% of CRC, is mostly MSS, and generally harbors mutations in DNA polymerase epsilon (POLe) [3]. POLe is the DNA polymerase that is largely responsible for leading strand synthesis during replication [4]. Most POLe mutations are located in the proofreading exonuclease domain, resulting in dramatically increased replication errors that ultimately contribute to large numbers of gene mutations [5]. Germline mutations in POLe are predisposed to CRC [6].

The exploration of large cancer genome datasets has revealed that tumors are characterized by hundreds to thousands of mutations, altering the functions of multiple genes [7]. However, not all mutations cause cellular transformation. In fact, most appear to be passenger, and may be regarded as background noise resulting from the genomic instability that occurs as cells transition from quiescent to transformed [8]. Although each independent mutation has little effect on cellular transformation, an accumulation of passenger mutations may also contribute to cancer evolution, and their role is being actively explored [9,10,11,12,13].

Those mutations known to have a direct effect on cellular transformation have been termed “driver”. Driver genes have also been identified [14], but there is a distinction between driver genes and driver mutations. Driver genes are highly mutated in nearly all cancers and generally regulate processes related to promoting cellular homeostasis (e.g., cell division, immunity, etc.). Various methods including mutation recurrence (e.g., frequency in cancer patients) and gene function have been used to classify these genes as “driver” [15,16,17,18]. Integrative Oncogenomics has used this information to compile a list of pan-cancer driver genes [14,19]. As expected, POLe is listed as a mutational cancer driver gene.

Mutations such as amino acid substitutions can also be classified as driver or passenger. As expected, driver mutations occur primarily in driver genes regulating the cell cycle, cell proliferation, DNA damage repair, immune surveillance, cell adhesion, metastatic development, and other cancer specific processes [20,21]. However, even within driver genes not every mutation has an equal impact on cancer progression, and computational programs have been developed to assess or predict the impact of mutations at any residue within the coding sequence of a protein.

Because it is ultimately mutations that lead to cancer, driver mutations can also be found in the coding regions of non-driver genes. This means that although a certain gene is not frequently mutated in cancer cells, certain mutations may significantly change the function of the gene to drive cancer. For example, an analysis of breast and colorectal cancers has shown that they are characterized by a multitude of mutations in genes that should not be frequently mutated in cancer cells, in addition to a handful of highly mutated genes [22]. CHASMPlus is one artificial intelligence algorithm that predicts driver mutations based on both frequency and functional significance [23]. Other algorithms also exist [15].

Most POLe mutations have been previously characterized [6,24,25,26,27,28,29,30,31]. The landscape of colorectal cancers with POLe mutations has also been previously reported on [6,25,26,30,31,32,33,34,35,36]. It has also been shown that POLe mutations tend to associate with mutations in other driver genes that promote cellular transformation and immortalization [3,36,37,38,39,40]. In this report, we re-analyzed cancer genomes deposited in COSMIC [41] or cBioPortal [42,43] to identify driver mutations in other genes that co-occur with POLe mutations. The goal was to use the CHASMPlus algorithm to classify every mutation that co-occurs with POLe mutations, regardless of whether it occurs within driver or non-driver genes.

## 2. Results and Discussion

### 2.1. POLe Mutation Spectrum in Human Cancers

Complete POLe mutation data from COSMIC and cBioPortal were first parsed into coding and non-coding data. Although most mutations reported by both sites are from similar samples, there are some differences: (1) only COSMIC reports synonymous mutations, (2) only COSMIC reports mutation zygocity, and (3) cBioPortal reports more samples than COSMIC. Thus, for a comprehensive analysis, we interrogated both sites (Supplementary Table S1).

**Figure 1:**
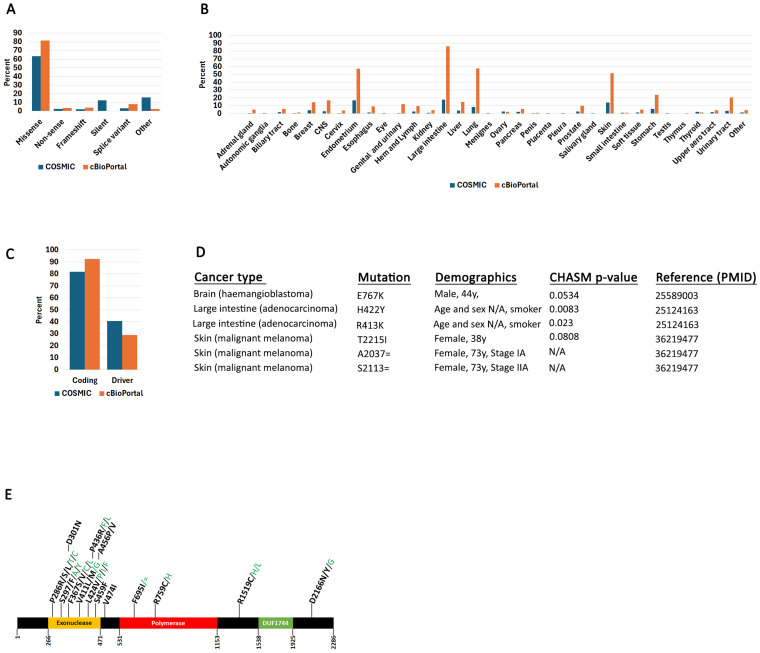
Polymerase epsilon mutation distribution in human cancers. (**A**). POLe mutation types reported in COSMIC and cBioPortal. “Other” refers to any other mutation that does not fall into the other categories and includes intronic and 5′ and 3′ UTRs. (**B**). Tissue distribution of mutations from (**A**). This distribution includes all mutations. (**C**). Percent COSMIC coding mutations (out of total mutations) and driver mutations (out of coding mutations). (**D**). Demographics of POLe homozygous mutations as reported in COSMIC. (**E**). Position of POLe high frequency mutations as reported in COSMIC. The bold black letters represent driver mutations, while the green letters represent other substitutions at the same position that CHASM did not identify as driver. Complete data in Supplementary Table S1.

Coding mutations occur within the translated regions of the gene (exomes), while non-coding mutations include 5′ and 3′ UTRs and intronic regions. The coding mutations were further parsed into missense, non-sense, frameshift, silent, splice variants, or other (Figure 1A). The “other” category includes InDels and other complex mutations. Most reported POLe mutations are missense. Synonymous mutations (silent) reported in COSMIC represent 12.1% of all mutations (including non-coding). Synonymous mutations are generally not thought to affect protein function. However, recent evidence suggests that they can also affect translation, splicing, and even transcription [44], but in this report, we focused only on characterizing non-synonymous coding mutations. Both COSMIC and cBioPortal report POLe mutations in nearly every cancer type (Figure 1B). As expected, the endometrium and large intestine have more POLe mutations than other tissues. cBioPortal also reports more mutations in lung and skin than COSMIC.

We used the CHASMPlus algorithm to analyze all reported POLe mutations. The algorithm generates a probability value, and we considered all mutations with a *p*-value below 0.05 to be “driver”. This analysis showed that a higher percentage of coding mutations are characterized as driver in the COSMIC than the cBioPortal dataset (Figure 1C). The reason for this is most likely due to the different sources used between the two sites.

Information on mutation zygosity from COSMIC is only available for 17.3% of samples (including coding and non-coding): 585 mutations (17%) are heterozygous and 12 mutations (0.3%) are homozygous. Of the 12 homozygous mutations, four occur in coding regions and two are synonymous (Figure 1D). A E767K mutation was reported in a 44-year-old male with brain hemangioblastoma [45]. Two large intestine adenocarcinoma patients (age and sex not reported) were characterized by two homozygous mutations (H422Y and R413K) [26]. These mutations are reported by COSMIC as two different samples from the same study, but it is not clear if they occur in the same patient. Finally, a 38-year-old female with skin malignant melanoma had a T2215I mutation [46]. Only the H422Y and R413K mutations that are in the POLe exonuclease domain (Figure 1E) were characterized as driver (CHASM *p*-value below 0.05). Two synonymous mutations were also identified in the same patient (73-year-old female) analyzed at two different time intervals (stages IA and IIA) [46]. The limited availability of mutation zygosity makes it hard to make any meaningful conclusions about the impact of these mutations on POLe function (e.g., whether mutations are recessive or dominant).

We next identified all high frequency mutations reported for POLe in COSMIC; we here define a mutation as “high frequency” when it occurs in four or more samples, based on previous algorithms used to identify hotspots [47,48,49,50]. Note, that high frequency mutations are not necessarily driver, although for POLe many are (see next section). We mapped these mutations on a POLe cartoon figure showing the positions of the protein domains (Figure 1E). POLe is characterized by an exonuclease proofreading domain at its N-terminus, a polymerase (enzymatic domain) in the center of the protein, and a DUF1744 domain of unknown function at its C-terminus [51,52]. As previously reported, most high frequency mutations cluster in the exonuclease domain [26,53]. Not unexpectedly, many of these mutations have driver potential.

A recent cryo-EM structure of human POLe as part of the replisome was recently solved, but only residues 1317–1926 were resolved in the catalytic subunit [54]. Thus, AlphaFold 3 was used to model the full-length protein bound to DNA, Mg^2+^, and Cl^−^ ions. This model was aligned to the partial cryo-EM human structure and an X-ray structure of *S. cerevisiae* POLe [55] (Supplemental Figure S1). Alignment of the human structural model with POLe from *S. cerevisiae*, which has 43.4% sequence identity, (PDB 4M8O) showed that DNA in the AlphaFold model aligns with what was experimentally observed in *S. cerevisiae*. Seven of the eight exonuclease site mutated residues (L424, S459, P286, F367, S297, A456, and P436) were found to be located within 12 Å of the AlphaFold predicted magnesium binding site. Coordination with Mg^2+^ is required for catalysis, and the mutation of residues around this metal ion binding site could prevent activity [55,56]. A previous report showed that F367 is highly conserved and A456 is somewhat conserved [57]. The L424 residue has been shown to be important for proofreading in *S. cerevisiae* [6]. The two mutated residues (F695 and R759) within the polymerase domain are located near the exterior of the protein and are not predicted to directly impact DNA binding. Residue R1519 near the DUF1744 domain is not located near protein–protein interfaces according to the cryo-EM structure.

### 2.2. POLe-Associated Mutations in Colorectal Cancers

The POLe gene is a driver of colorectal and endometrial cancers, and mutations occur most frequently in these cancers [30,58,59]. We wanted to investigate the mutation spectra of colorectal cancers that have POLe mutations. We first partitioned files with colorectal cancers or all cancers into three groups: (1) samples with POLe driver mutations, (2) samples with POLe non-driver mutations, and (3) samples with WT POLe (no mutation). The driver potential for POLe mutation in these two files was determined using CHASMPlus (Supplementary Table S2) [23]. Then, we analyzed the mutations that occur in other genes in these three groups for each file. This showed that for colorectal cancers, a driver POLe mutation is more likely to associate with mutations in other genes that are unlikely to have driver potential compared to other cancers (Supplementary Figure S2). However, this analysis also revealed that colorectal cancers are generally characterized by fewer driver mutations in all three groups (POLe driver, WT, or passenger background) compared to all cancers. This analysis should be taken with the caveat that the colorectal cancers were not partitioned into the three groups: non-hypermutated, hypermutated, and ultramutated. COSMIC does not report these characteristics, so it is entirely possible that there are confounding factors.

**Figure 2:**
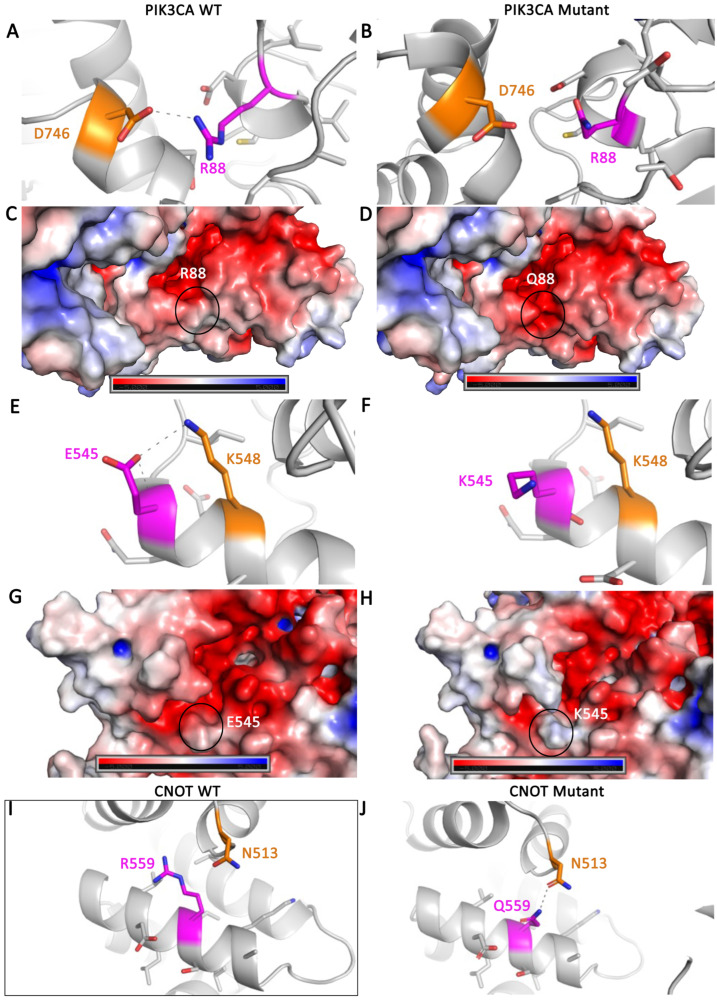
Protein structure modeling of select PIK3CA and CNOT mutations. The PIK3CA structure (PDB ID: 2RD0) is shown as a gray cartoon with (**A**) the WT R88 side chain in magenta interacting with the side chain of D476 (shown in orange) through a salt-bridge shown with a gray dashed line, (**B**) the mutant Q88 side chain in magenta, (**E**) the WT E545 side chain in magenta interacting with the side chain of K548 (shown in orange) through a salt-bridge shown with a gray dashed line, and (**F**) the mutant K545 side chain in magenta. The electrostatic surface potential of PIK3CA, where a negative charge is shown in red, neutral in white, and positive charge in blue, with the residue of interest circled for (**C**) WT R88, (**D**) mutant Q88, (**G**) WT E545, and (**H**) mutant K545. The CNOT AlphaFold model is shown as a gray cartoon with (**I**) the WT R559 side chain in magenta and (**J**) the mutant Q559 side chain interacting with the side chain of N513 (shown in orange) through a hydrogen bond shown with a gray dashed line.

To understand whether mutation hotspots occur in any of these genes, we searched for high frequency mutations in the genes identified in Supplementary Table S2. This analysis revealed four genes (PIK3CA, CNOT, FBXW7, CTNNB1) with hotspot driver mutations that co-occur with POLe driver mutations, POLe non-driver, or WT (Supplementary Figure S3). The mutations in these four genes were modeled computationally to determine their impact on protein structure. Each mutation was studied for their impact on tertiary structure interactions and electrostatic surface potential using PyMOL. In addition, the CUPSAT online tool was used to predict the impact on protein folding and stability (Table 1) [60]. CUPSAT reports a ΔΔG value that represents the difference in the folding of the wild-type and mutant ΔG values, where a negative ΔΔG is destabilizing and a positive ΔΔG is stabilizing. For the three PIK3CA mutations (R88Q, E545K, and H1047R), it was observed that both R88Q and E545K reduced polar tertiary structure interactions and changed the electrostatic surface potential of the protein (Figure 2A–H). The R88 residue helps to stabilize the packing of the ABD and kinase domain together, and the loss of this tertiary structure interaction is expected to impact the overall fold of the protein. The E545 residue is found in the helical domain and makes a tertiary structure interaction within the α-helix in which it is found. Mutation of the E545 residue is expected to have a smaller and more local impact on the protein structure than R88Q. The H1074R mutation did not affect change in tertiary interactions or the electrostatic surface potential. In addition, it was predicted by CUPSAT that the R88K mutation is significantly destabilizing to the protein structure, with a ΔΔG of −4.53 kcal/mol. The E545K and H1074R mutations had ΔΔG values that were less than 1 kcal/mol and thus did not significantly affect protein stability. The mutations in CNOT, FBXW7, and CTNNB1, shown in Supplementary Figure S3, were also computationally modelled onto their respective protein structures. The CNOT R559Q mutation was found to change the polar tertiary interactions (Figure 2I,J), but not the electrostatic surface potential or protein stability. None of the FBXW7 or the CTNNB1 mutations were found to affect polar interactions or electrostatic potential (Table 1). However, the FBXW7 R465Q/H mutations were predicted to destabilize protein structure, with ΔΔG values of −1.83 and −1.40 kcal/mol, respectively, and the R465C mutation was extremely stabilizing, with a ΔΔG of +7.88 kcal/mol. The CTNNB1 S45F mutation was also predicted to destabilize protein folding, with a ΔΔG of −1.87 kcal/mol.

To understand whether co-mutations between POLe and the genes identified here are likely to co-occur, we used the cBioPortal mutual exclusivity calculator. This analysis shows that, indeed, there is a high probability that mutations between POLe and the genes modeled here are likely to co-occur in human cancers (Supplementary Table S3). This suggests that there could be functional co-operation between POLe mutations and mutations in these genes to produce cellular transformation and immortalization.

Because POLe is a replicative polymerase, mutations in this gene produce specific nucleotide substitution signatures [61]. Consequently, we analyzed all nucleotide substitution patterns appearing in colorectal cancers in the background of POLe driver, POLe non-driver, and WT (Supplementary Figure S4, Supplementary Table S4). We did not find any mutation signatures that were strongly associated with POLe driver mutations. Previous analysis of human cancer identified specific POLe mutation signatures (Signature 10a–10d) [3,24,62]. These signatures were classified as follows: C>A and C>T for polymerase epsilon exonuclease mutations (Signatures 10a,b), and C>A for polymerase proofreading mutations (https://cancer.sanger.ac.uk/signatures/sbs/ (accessed on 16 July 2025)). Our analysis did not reveal this because we grouped together all POLe driver and non-driver mutations, and POLe driver mutations include exonuclease and proofreading activity mutations as well as other mutations in other domains. Thus, although specific POLe driver mutations can produce unique signatures, a pan-POLe mutation analysis does not reveal any patterns.

To further investigate the position of POLe mutations co-occurring with the other genes studied here, we stratified the data by POLe domains shown in Figure 1E. As expected, this analysis shows that most driver POLe mutations occur in the exonuclease and polymerase domain of the gene (Supplementary Table S5). However, this analysis does not suggest that a driver mutation is more likely than a non-driver mutation to associate with mutations in other genes or vice versa (Supplementary Table S5A,B). Thus, it appears that the genetic interaction between POLe driver mutations and mutations in other genes is complex (e.g., POLe mutations do not necessarily lead to purifying the selection of mutations in other genes).

### 2.3. Colorectal Cancer Differential Gene Expression Patterns in POLe Mutant Samples

**Figure 3:**
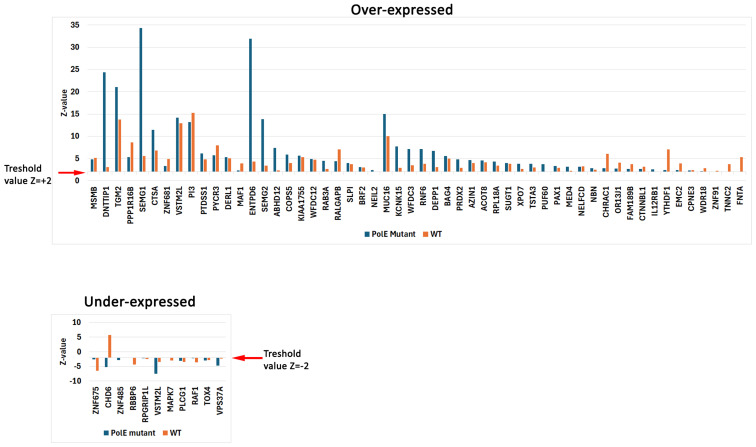
Expression levels of other genes in POLe mutated and POLe WT samples. The data is expressed as a Z-value, which is normalized to WT tissue. A Z-value between −2 and +2 is considered normal expression. A Z-value below −2 is under expressed and a Z-value above +2 is overexpressed. The +2 and −2 threshold values are indicated with red arrows, but changes in expression should be interpreted by comparing with WT control (e.g., VSTM3L is over-expressed in both POLe mutant background and in POLe WT background and is interpreted as no change between the two POLe genotypes). Note that this figure includes all data available for the indicated genes from COSMIC. However, certain genes show highly over-expressed values in POLe mutation background when compared to WT control (e.g., DNTTIP1, SEMG1, ENTPD6). However, this skew is not a general trend, but is produced by a few samples. Please see text for more in-depth description of these outlier samples. Complete data is found in Supplementary Table S6.

To investigate whether POLe mutations alter gene expression, we extracted genome-wide expression values (Figure 3, Supplementary Table S6). Gene expression values are only available for TCGA patient data and are provided as normalized Z-scores (see Materials and Methods Section 3). Overall, we did not identify any differential gene expression trends between POLe mutant and POLe wild-type samples. Although certain samples did show altered gene expression, most samples had gene expression within the normal range. For example, DNTTIP1 expression levels were reported in 12 samples, but only one showed high over-expression (Z = +24.391), while the expression levels in the other 11 were within the normal range (Z = +/−2).

Nevertheless, we investigated some samples with genes showing significantly altered differential expression profiles between POLe driver and POLe WT samples (Figure 3). We emphasize here that abnormal expression profiles in the genes described below are patient-specific and do not constitute a general trend in the cancers studied here. Rather, the general interpretation is that there do not appear to be changes in the expression of other genes in POLe mutant samples. Four genes were over-expressed in specific samples with POLe mutations (SEMG1, SEMG2, DNTTIP1, ENTPD6). SEMG1 and SEMG2 encode for Semonegelin 1 and 2, involved in spermatozoa function [63]. These two genes have been previously reported to be over-expressed in sessile serrated lesion colorectal cancers [64]. Both genes showed high over-expression patterns in an 80-year-old female with ascending colon adenocarcinoma. The same patient also showed under-expression of CHD6 (chromodomain DNA helicase binding 6), a gene involved in heterochromatin establishment and DNA double-strand break repair [65]. CHD6 expression is controlled by Wnt and EGF signaling pathways and may promote colon cancer [66]. DNTTIPI is a deoxnucleotidyl transferase terminal interaction protein that functions in gene transcription and cell division [67]. This gene has been flagged as over-expressed in certain colorectal cancers and is associated with poor survival [68]. ENTPD6 catalyzes extracellular nucleosides and is involved in platelet aggregation, smooth muscle contraction, and pain perception, among other functions [69]. Overexpression of this gene was identified in a 77-year-old male with colon ascending adenocarcinoma. To our knowledge, over-expression of this gene in colorectal cancers has not been previously reported.

### 2.4. Evidence of POLe-Specific Mutation Signatures from In Vivo Mutagenic Screens and Experiments

Although the purpose of this report was to analyze colorectal data deposited in COSMIC or cBioPortal, other studies have tested the impact of some of these mutations in human cells. For example, one report shows that certain POLe mutations (P286R, V411L, S459F) can produce specific genomic signatures and increase mutation burden [70]. Interestingly, other studies mapping co-occurring mutations using patient data also show an association between POLe exonuclease driver mutations and similar genes that we identified here (e.g., KRAS, PTEN, PIK3CA) [71]. Certain POLe germline mutations are also known to produce unique cancer signatures [6,72]. However, experiments directly testing the impact on the cellular transformation of co-occurring POLe and mutations in other genes is sparce. Indeed, as our data and other studies show, cancers are complex, and most have mutations in multiple genes that would be hard to reproduce in an experimental setting.

## 3. Materials and Methods

### 3.1. Data Accession and Processing

Files with POLe mutations were downloaded from COSMIC [41] and cBioPortal [42,43,73]. For COSMIC, the data were downloaded as .csv files and contain both primary cancers (patients) and cell lines (accessed on 6 March 2022). cBioPortal data was accessed using cBioPortal’s public API (https://www.cbioportal.org/api/swagger-ui/index.html?urls.primaryName=public (accessed on 7 July 2024)) and Python 3.13, and the main website (https://www.cbioportal.org/ (accessed on 7 July 2024)). The data set were selected using the “Curated set of non-redundant studies” tab, but with all pediatric studies removed. Data were downloaded as .csv files and contained primary cancer data only. The API and Python 3.13 were used to retrieve specific data (POLe mutations, POLe-associated mutations, and non-POLe-associated mutations) from the set.

### 3.2. Determination of Mutation Driver Potential

Using the/api/mutations/fetch function of the cBioPortal API and Python 3.13, the samples from the selected cBioPortal datasets were filtered for those with POLe mutation(s). To classify these mutations as driver or non-driver, we used the OpenCravat (https://run.opencravat.org (accessed on 7 July 2024)) CHASM AI algorithm (CHASMPlus) [23]. The algorithm generates probability values for all mutations; mutations with probability values below 0.05 were considered driver.

### 3.3. TCGA Expression

COSMIC makes available gene expression levels for certain TCGA samples. These expression profiles are from microarray analysis or RNA-seq and are reported as a Z-value. Z-values are normalized to non-tumor tissue and should be interpreted as follows: values above Z = +2 represent over-expression, while values under Z = −2 represent under-expression. A value between −2 and +2 is interpreted as normal expression [74,75].

### 3.4. Pan-Cancer Analysis of POLe Mutations

We carried out a pan-cancer analysis of POLe co-occurring mutations. For this analysis, we used the cBioPortal repository. Using the /api/mutations/fetch function of the cBioPortal API and Python 3.13, the patient samples from the selected cBioPortal datasets were filtered for those with POLe mutation(s). From those samples, the number of those that occurred in the POLe mutation sample set for each mutant gene was counted, and data on the 233 genes that had 200 or more mutations were analyzed, while the rest was filtered out. The data is partitioned into mutations occurring in a POLe driver background, POLe non-driver background, or POLe WT background.

### 3.5. Protein Structure Analysis

An AlphaFold 3 model of human POLe (AF-Q07864-F1-v4) was generated [76] bound to a DNA segment (strand 1: TAACCGCGTTC and strand 2: CTCTTGAACGCGGTA), 2 Cl^−^ ions, and 1 Mg^2+^ ion. The AlphaFold model was aligned to an X-ray structure of S. cerevisiae POLe (PDB ID: 4M8O) [77] using the PyMOL Molecular Graphics System (version 3.0.3). Mutations were generated using the mutagenesis function in PyMOL, using a published X-ray structure of PIK3CA (PDB ID: 2RD0) [78] and AlphaFold models of CNOT1 (AF-A5YKK6-F1-v4), FBXW7 (AF-Q969H0-F1-v4) and CTNNB1 (AF-P35222-F1-v4) [76]. The APBS (Adaptive Poisson-Boltzmann Solver) PyMOL plug-in was used to generate electrostatic surface potentials. Structures were uploaded to the CUPSAT server to generate protein stability metrics [60].

All figures were made in Photoshop.

## 4. Conclusions

This analysis highlights the importance of POLe mutations in driving cellular transformation and immortalization in human cancers. We show here that colorectal cancers harbor driver mutations in both driver and non-driver genes. Similarly to previous studies [22], multiple genes that are not classified as driver contribute to the transformation of colon cancer cells. Additionally, although POLe is a major driver gene, a driver POLe mutation does not cause accumulation of driver mutations in other genes.

## Figures and Tables

**Table 1:** Results from protein structural modeling of POLe co-occurring mutations.

Protein	Mutation	Tertiary Structure Interaction Changes	Electrostatic Surface Potential Changes	ΔΔG Value (kcal/mol)
PIK3CA	R88Q	Yes, decreased	Neutral to negative	−4.53
	E545K	Yes, decreased	Negative to neutral	+0.08
	H1074R	No	Negative to partial negative	−0.56
CNOT	R559Q	Yes, increased	No change	−0.08
FBXW7	R465Q	No	No change	−1.83
	R465H	No	No change	−1.40
	R465C	No	No change	+7.88
CTNNB1	S45F	No	No change	−1.87
SF3B1	R196Q	No	Positive to negative	−0.23
	R315Q	No	Positive to neutral	+0.54
CHD4	R1338I	No	No change	−2.31
CHD8	R546C	No	Positive to partial positive	−0.37

## Data Availability

The original contributions presented in this study are included in the article/Supplementary Materials. Further inquiries can be directed to the corresponding authors.

## Supplementary Materials

The supporting information can be downloaded at: https://www.mdpi.com/article/10.3390/ijms26157208/s1.
