# Supplementary material for: Comprehensive Analysis of Human Colorectal Cancers Harboring Polymerase Epsilon Mutations
Source: Int J Mol Sci. 2025 Jul 25;26(15):7208. doi: 10.3390/ijms26157208 (PMC12347369; doi:10.3390/ijms26157208)

Figure S1. POLe Structures. A) Alignment of human POLe PDB 7PFO (magenta) with *S. cerevisoae* POLe PDB 4M8O (cyan) and a human POL3 AlphaFold model (gray). Bound dsDNA is shown in orange and chloride and magnesium ions are shown wtih green spheres. B) Domain coloring of the human POLe AlphaFold model with the DUF1744 domain colored green, the exonuclease domain colored yellow, and the polymerase domain colored red. C) Position of the mutated residues is shown with magenta sitcks in relation to the magnesium ion binding site. D) AlphaFold predicted local distance difference test (pLDDT) metrics are displayed on the human POLe structure with the specified colors. The interface predicted template modeling (ipTM) score is 0.86 and the predicted template modeling (pTM) score is 0.74.

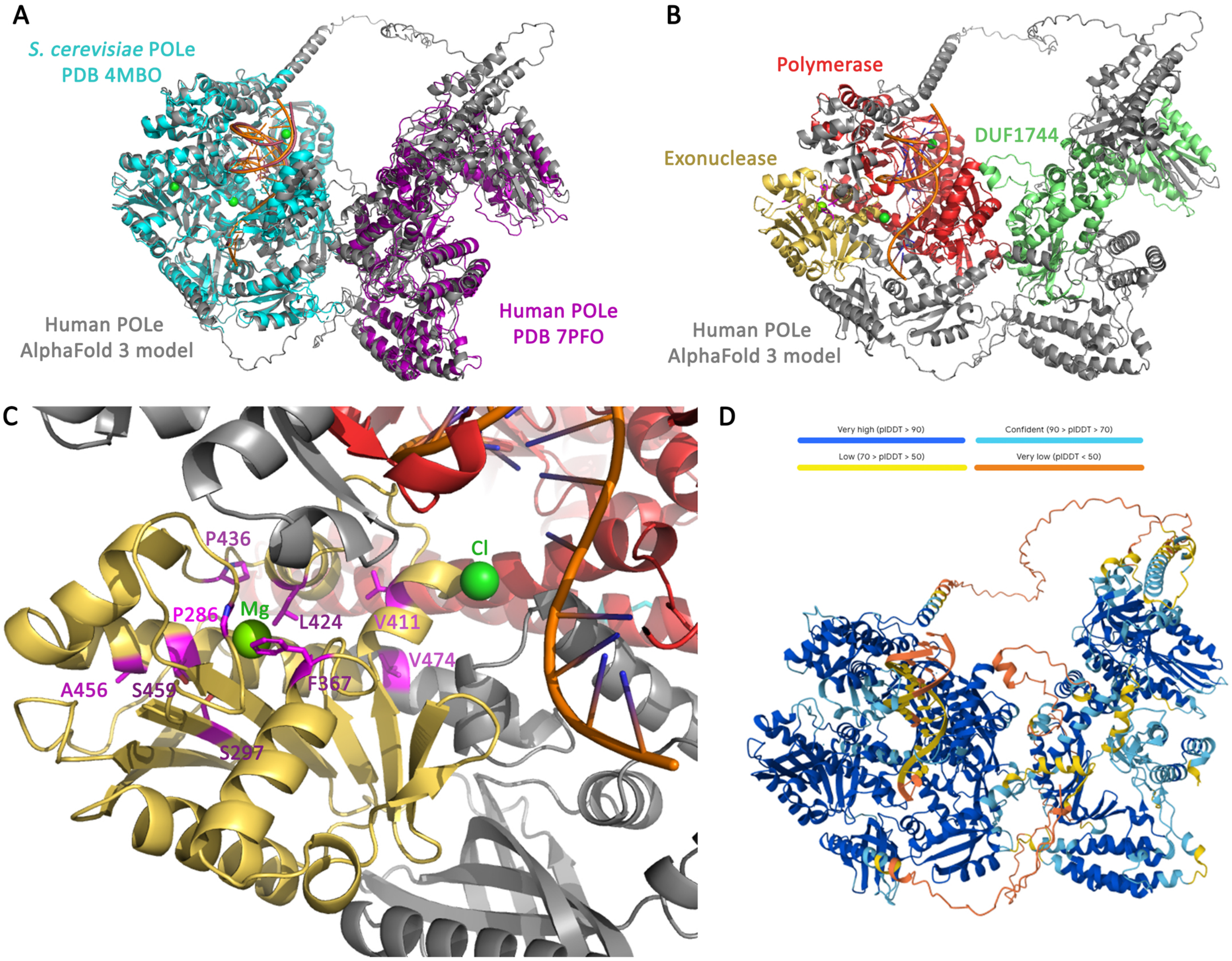

Supplement: Supplementary file 1 [file ijms-26-07208-s001.zip › Fig. S1.pdf]
