# Supplementary material for: Comprehensive Analysis of Human Colorectal Cancers Harboring Polymerase Epsilon Mutations
Source: Int J Mol Sci. 2025 Jul 25;26(15):7208. doi: 10.3390/ijms26157208 (PMC12347369; doi:10.3390/ijms26157208)

Supplementary Figure 3. High frequency mutations co-occurring with polE mutations in colorectal cancers. Maps with high frequency mutations. Red- occurring in driver POLe background, black-occurring in non-driver POLe background, blue-occurring in WT background. Mutations with multiple colors occur in several backgrounds.

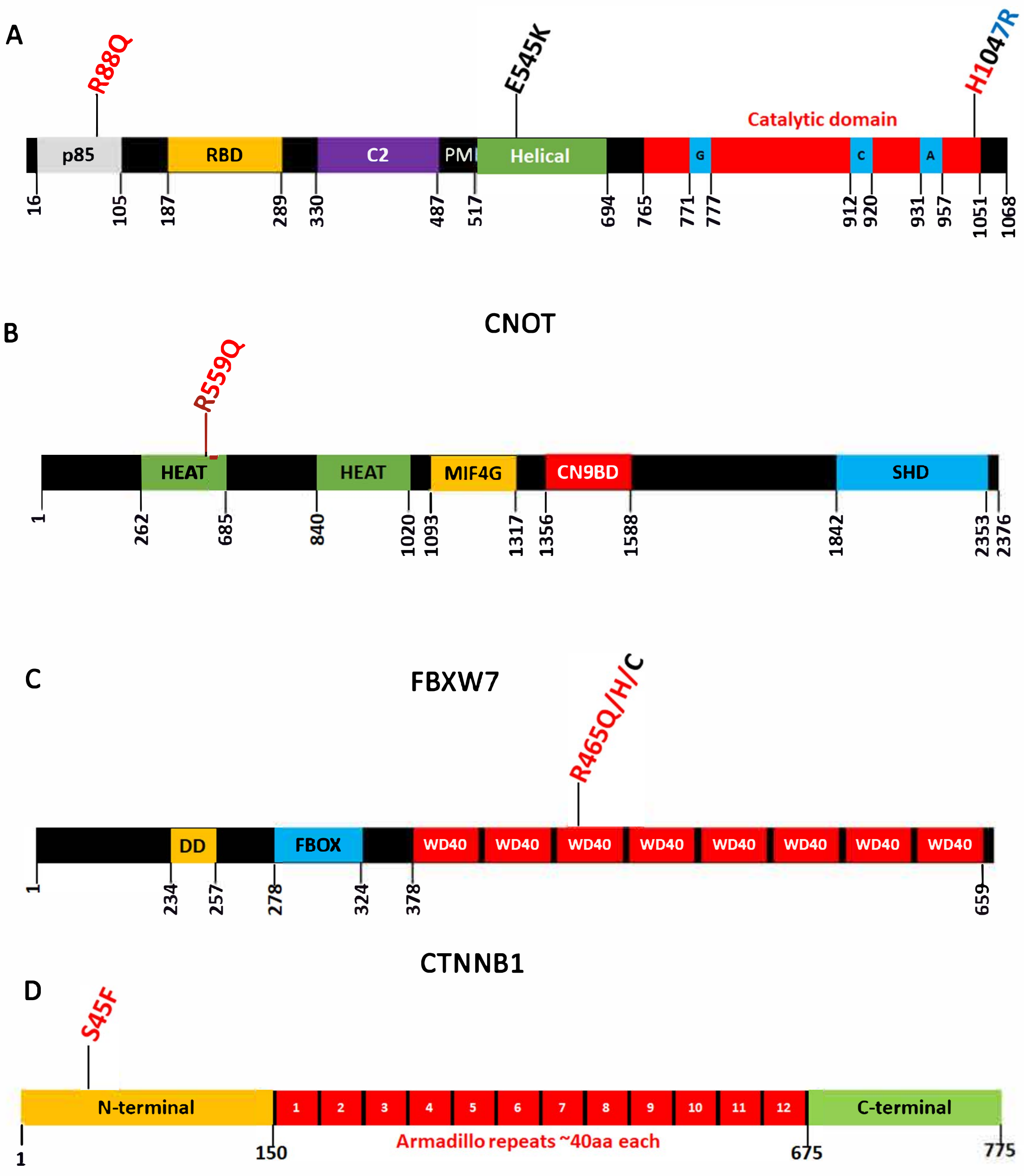

Supplement: Supplementary file 1 [file ijms-26-07208-s001.zip › Fig. S3.pdf]
