# Supplementary material for: Comprehensive Analysis of Human Colorectal Cancers Harboring Polymerase Epsilon Mutations
Source: Int J Mol Sci. 2025 Jul 25;26(15):7208. doi: 10.3390/ijms26157208 (PMC12347369; doi:10.3390/ijms26157208)

Supplementary Figure 2. Driver potential of other mutations occurring in the background of POLe WT, driver and passenger mutations.

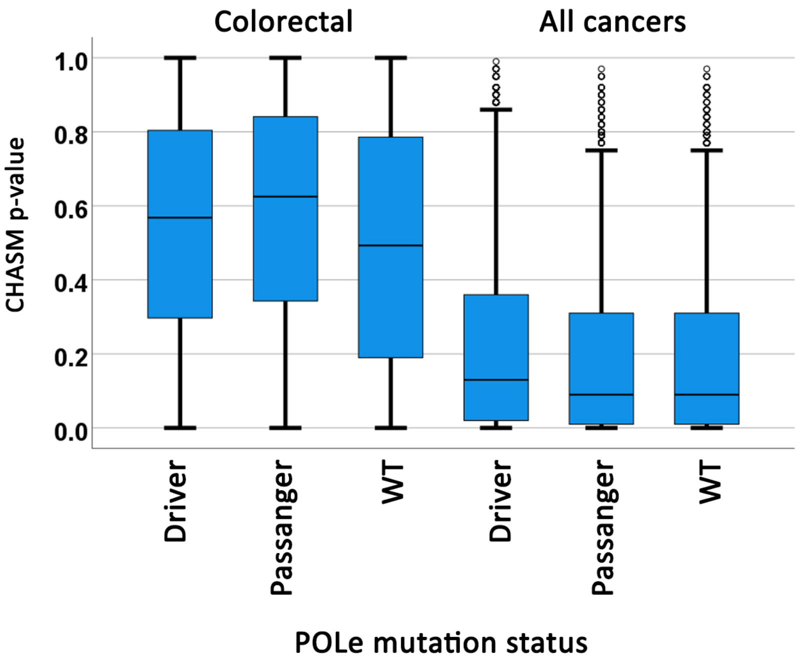

Supplement: Supplementary file 1 [file ijms-26-07208-s001.zip › Fig. S2.pdf]
