# Supplementary material for: Comprehensive Analysis of Human Colorectal Cancers Harboring Polymerase Epsilon Mutations
Source: Int J Mol Sci. 2025 Jul 25;26(15):7208. doi: 10.3390/ijms26157208 (PMC12347369; doi:10.3390/ijms26157208)
Supplement: Supplementary file 1 [file ijms-26-07208-s001.zip › Table S3.pdf]

Supplementary Table S3. Probability of co-occurring mutations between POLe and the indicated genes.

Gene (Co-occurring with POLe)	Log_2	p-value	q-value	Tendency
PIK3CA	1.631	<0.001	<0.001	Co-occurrence
CNOT	2.671	<0.001	<0.001	Co-occurrence
FBXW7	2.617	<0.001	<0.001	Co-occurrence
CTNNB1	2.029	<0.001	<0.001	Co-occurrence
SF3B1	2.360	<0.001	<0.001	Co-occurrence
CHD4	>3	<0.001	<0.001	Co-occurrence
CHD8	>3	<0.001	<0.001	Co-occurrence
